# Utilization of calcium-rich groundwater desalination concentrate in aquaculture

**DOI:** 10.1016/j.heliyon.2023.e16140

**Published:** 2023-05-15

**Authors:** Sivan Klas, Idan Rom, Yakov Peretz

**Affiliations:** a Department of Biotechnology Engineering, Braude College of Engineering, Karmiel 2161002, Israel; b Department of Civil and Environmental Engineering, Technion IIT, Haifa 3200003, Israel; c Algae-Smart Ltd., Algae-Fish Lab, Meir Shfeya, 3080600, Israel

**Keywords:** Desalination, Concentrate, Brine, European seabass, Aquaculture

## Abstract

Global production of desalinated water increased greatly over the past three decades. Brackish water desalination is energetically favorable compared with seawater desalination, but high treatment costs and negative environmental impact of the concentrate byproduct hinders its development in semi-arid regions. The present study assessed key considerations associated with potential commercial aquaculture in high-flowrate calcium-rich groundwater desalination concentrate. European seabass (*Dicentrarchus labrax*) fingerlings, weighing 20–40 g were cultivated in brackish water (control), raw concentrate, and partially softened concentrate under flow-through conditions. Aside from two disease-related mortality events, fish survival exceeded 92% during 70 days of cultivation in all water types. The highest average growth rate of 0.26 g d^−1^ was obtained in the partially softened concentrate, which was 27% and 83% higher than in the raw concentrate and in the control, respectively. Substantial mineral precipitation on equipment and minor gill damage were observed in fish tanks receiving raw concentrate, projecting serious operational issues under commercial application. Preliminary aeration-softening of the concentrate relieved CO_2_ supersaturation and prevented precipitation issues. Several implementation options in a case study fish farm predict commercial and environmental feasibility in specific locations.

## 1 Introduction

Increasing population and pollution of water resources increased water scarcity around the world [1,2]. Correspondingly, the production of desalinated water increased greatly over the past three decades [2]. Due to the relatively low salinity of brackish water (2,000–10,000 mg L^−1^), its desalination requires up to 16% of the energy required for seawater desalination by either electrodialysis [3] or reverse osmosis [4]. Currently, desalination of brackish water accounts for 21% of the global desalination industry output [2]. However, the concentrate byproduct (also termed brine, reject water and retentate), pose a significant environmental threat upon inland disposal [1,2,5,6]. Disposal options of brackish water desalination concentrate (BWDC) include surface water discharge, sewer discharge, deep-well injection, evaporation and land application [1]. The costs associated with disposal are generally low, but in arid and semi-arid locations, where land and natural water sources are under constant salination risk, these options are often not permitted or not feasible [1,6]. Another BWDC management approach is its treatment with the aim of zero liquid discharge. The following stages are usually involved: preconcentration, evaporation, crystallization, and solid disposal or processing [1]. Although BWDC treatment allows a significant increase in water recovery and waste reduction, significant challenges were reported, including lack of commercial maturity, high capital costs, accumulation of operational costs at each treatment stage, high energy demand, need for expensive materials, and overall process complexity [1]. Due to the absence of cost effective, environmentally friendly, and mature technologies for BWDC treatment and disposal, the development of highly needed brackish groundwater desalination plants in Israel is often hindered.

The purpose of the present work is to explore the feasibility of BWDC utilization through aquaculture in an environmental manner. This possibility attracted considerable interest from fish farmers in Israel who face quality water shortage, and as this new water source may allow cultivation of profitable euryhaline fish species at constant temperature of around 22 °C with low risk of pathogen introduction [7]. The premise of the current work is that in coastal locations, BWDC may be discharged to the sea after harvest, while in inland locations, BWDC may be discharged, together with the entire fish farm's water volume, into floodwater streams during rainy wintertime. BWDC and fishpond salinities in Israel are typically around 15 dS m^−1^ and <5 dS m^−1^, respectively, while permissible fishpond discharge salinity during wintertime is up to 12.5 dS m^−1^ (10,000 mg L^−1^), depending on the receiving stream [8]. Therefore, in certain locations the proposed strategy (BWDC utilization through aquaculture) can safely comply with environmental regulations.

The literature regarding the feasibility of fish cultivation in BWDC is, however, surprisingly limited. Sànchez et al. [6] reported that Koina tilapia (*Oreochromis* sp.), well adapted to elevated water salinity, was cultivated in two types of BWDC, having salinities of 11.38 mS cm^−1^ and of 9.82–13.38 mS cm^−1^, for 153 and 170 days respectively, in the semi-arid region of Brazil. Survival rates reached 80% and of 94.7%, respectively. Cultivation was, however, conducted under relatively low concentrate flowrates of 1–2.5 m^3^ h^−1^, not typical to large scale desalination plants. Furthermore, water aeration was not employed, in contrast with commercial intensive cultivation. When using calcium-rich BWDC, concerns were raised regarding CaCO_3_ precipitation on external fish organs and on submerged equipment following such aeration [7]. We have recently reported [7] that European seabass (*Dicentrarchus labrax*) demonstrated 100% survival during 3 months of cultivation in both calcium rich BWDC (∼2,200 mg L^−1^ as CaCO_3_) and in partially-softened BWDC (SBWDC). However, the fish were grown in a nearly closed system, not typical to commercial intensive cultivation, where daily water exchange rates exceed 10% [9]. Under normal water exchange rate, calcium load is expected to be dramatically higher, and the extent of potential calcium precipitation increase accordingly. The present work presents the first controlled investigation of European seabass cultivation under flow-through conditions (high water exchange rate) in both calcium rich BWDC and in SBWDC. The results were used to assess several implementation options of the proposed approach in a case study fish farm.

## 2 Experimental

**Fig. 1:**
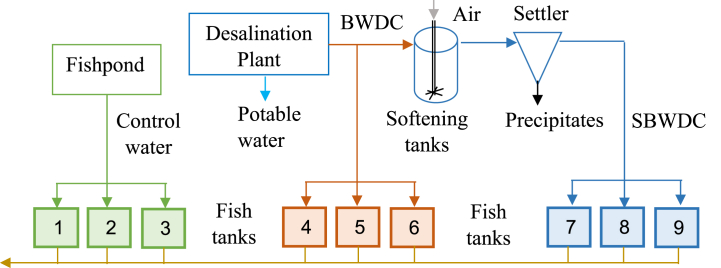
Schematic description of the experimental system.

The experiment was conducted at Kfar Masaryk fish farm in northern Israel, and in the vicinity of Kfar Masaryk desalination plant, which desalinates approximately 8 million m^3^ y^−1^ brackish groundwater at 83% recovery rate. The BWDC is currently injected into 180 m deep saline well through a main BWDC disposal line, from which BWDC for the experiment was obtained. European seabass was cultivated in three water types: BWDC, SBWDC and brackish water obtained from an operational fishpond as control. The use of the latter water source was due to the absence of a better alternative (e.g., fresh brackish water) in that location. Three duplications of each water type were employed, as shown in Fig. 1.

A separate submerged pump was used for each water type to attain desired flow rates into each fish tank. The flowrates were approximately 3.3 m^3^ d^−1^ and 6.6 m^3^ d^−1^ for the concentrate receiving fish tanks and for the control fish tanks, respectively. The control water contained relatively high ammonium and nitrite concentrations, and higher flowrate into these tanks was thus employed to minimize their concentrations. All fish tanks contained 0.7 m^3^ water, resulting in 5 h hydraulic retention time (HRT) or 4.7 water replacements per day, in the concentrate fed fish tanks. These relatively high exchange rates resemble “flow-through” cultivation systems intended to maintain toxic metabolic wastes at adequate levels [10]. Softening (i.e., calcium removal) was attained by CO_2_ stripping through aeration, following Eq. (1) [7].

(1) $$Ca^{+2}\left(aq\right)+2HCO_{3}^{-}\left(aq\right)\to CaCO_{3}\left(s\right)+CO_{2}\left(g\right)+H_{2}O\left(l\right)$$

Aeration of the fish tanks and of the softener was supplied by a 1.5 kW regenerative blower (1RSC, Renova), at a total estimated flowrate of about 130 m^3^ h^−1^. BWDC softening was carried out in two 1.5 m^3^ tanks connected in series. Overall, HRT in the softening tanks was approximately 7 h. Air was injected into the softening tanks through a vertical stainless-steel pipe to which six horizontal 1 cm diameter outlets were connected to its lower end, which was placed at the bottom of the softening tanks (Fig. 1). The relatively large air outlets prevented frequent clogging that occurred when using smaller outlets. Mixing was attained by the air bubbles only, as attempts to improve mixing and recycle solids failed due to pump clogging by precipitates. SBWDC from the softening tanks flowed into two 0.25 m^3^ conical tanks, connected in series, used for gravitational solids separation. The precipitate free SBWDC then entered the fish tanks, as depicted in Fig. 1.

Before experiment start, approximately 800 European seabass fingerlings, weighing approximately 5 g, were placed in fish tanks 1–9 and were cultivated in control water for approximately 3 weeks. Following this stage, fish tanks 4–9 were acclimatized in a mixture of about 50% control water and 50% raw BWDC or SBWDC for nearly 4 weeks. The fish were then weighed, counted, and transferred to attain 90 ± 2 fingerlings per tank. The corresponding fish density was approximately 0.65 kg-fish m^−3^. After two days, control water supply to tanks 4–9 was halted and thereafter (experiment start) tanks 4–6 received BWDC only, while tanks 7–9 received SBWDC only. Tanks 1–3 continued receiving control water only. The fish were fed once or twice a day (excluding weekends) until satiation by Zemach 4992 pellets, containing 52% and 18% protein and fat, respectively.

Fish were indirectly weighed several times during the experiment by measuring water volume change after addition of 9–15 fingerlings to a partially filled 1 L glass measuring cylinder. At the end of the experiment, all fish were counted and weighed.

Water quality parameters were regularly monitored using portable meters for temperature and pH (pH 450, EUTECH), oxygen (OxyGuard Polaris), and electrical conductivity (EC 450, EUTECH). Samples for nitrogen species determination were acidified by 6 M HCl solution immediately after collection and filtered (FT-3-328-090, Munktell) before analysis. Nitrate, nitrite and total ammoniacal nitrogen (TAN) concentrations were measured colorimetrically using appropriate Merck Spectroquant® test kits. Calcium hardness (CaH) was measured by EDTA titration [11]. Alkalinity with respect to H_2_CO_3_* equivalence point (Alk) was determined following Gran method [12]. Dissolved CO_2_ concentration was calculated from pH and alkalinity data, after correcting the equilibrium constants for temperature and ionic strength effects [13]. At the beginning and at the end of the experiment, 3 fish from each tank were collected before feeding, placed in plastic bags filled with approximately 2 L water and 3 L air, and delivered alive for pathological and histological examination at the central fish health laboratory at Nir David, Israel. Upon arrival, the fish were sedated using phenoxyethanol and subjected to external visual inspection followed by microscope examination of samples taken from the body, gills, and internal organs.

## 3 Results and discussion

### 3.1 Softening system performance

The softening system operation was usually steady and maintenance free except for precipitate removal, which was difficult due to frequent pipe clogging. Table 1 presents average values of selected water quality parameters in the softener inflow (BWDC) and outflow (SBWDC). A more comprehensive BWDC composition at the same location was recently reported [13].

**Table 1:** Average (standard deviation) values of water quality parameters in the softener inflow (BWDC) and outflow (SBWDC) during the experiment (N = 20).

	CaH	Alk	pH	Temp	EC	CO_2_
	mg CaCO_3_ L^−1^			^o^C	dS m^−1^	mg L^−1^
BWDC	2231 (147)	1669 (76)	7.6 (0.1)	26.2 (2.2)	15.2 (0.5)	37 (3)
SBWDC	939 (195)	461 (60)	7.7 (0.1)	26.1 (2.2)	14.2 (0.4)	10 (3)

The inflow values of CaH, Alk and EC presented in Table 1, are in good agreement with previously reported data [13]. The average temperature of the raw concentrate was 4.2 °C higher than previously reported, probably because the BWDC flowed from the main BWDC disposal line to the softening system through a 150 m long black plastic pipe subjected to solar radiation and exposed to characteristic ambient temperatures of 20–32 °C. The similar pH values of the raw and softened concentrate supported the premise that the chemical reaction followed Eq. (1). Recommended CO_2_ threshold concentrations for aquaculture in warm water are 20–40 mg L^−1^ [14]. Table 1 thus suggests that CO_2_ concentration in the BWDC is potentially harmful to fish and that preliminary aeration can resolve this issue. Ammonia and nitrite are toxic to fish at concentrations as low as 1 mg L^−1^ [15]. However, ammonia and nitrite values in the BWDC were within the detection error (<0.1 mg L^−1^). The values of CaH, Alk and EC in the SBWDC were lower than in the BWDC due to calcium and bicarbonate removal during the softening process (Eq. (1)). Table 1 indicates that calcium and alkalinity removal reached 1.25 (±0.05) and 1.19 (±0.03) g L^−1^ – CaCO_3_, respectively. This is in good agreement with Eq. (1), which predicts equal alkalinity and calcium removal, since alkalinity is removed by carbonate precipitation, but not by gaseous CO_2_ release [16]. It is noted that after 24 h aeration of concentrate from the same source, the solution reached equilibrium and corresponding calcium and alkalinity removal reached 1.6 g L^−1^ – CaCO_3_ [13]. Thus, minor CaCO_3_ precipitation was anticipated in the fish tanks receiving SBWDC, while major CaCO_3_ precipitation was anticipated in fish tanks receiving BWDC. It can be concluded that the softening system was efficient but used relatively large water volume and involved hurdles of precipitates separation. Better design is required for commercial implementation.

### 3.2 Water quality in the fish tanks

**Fig. 2:**
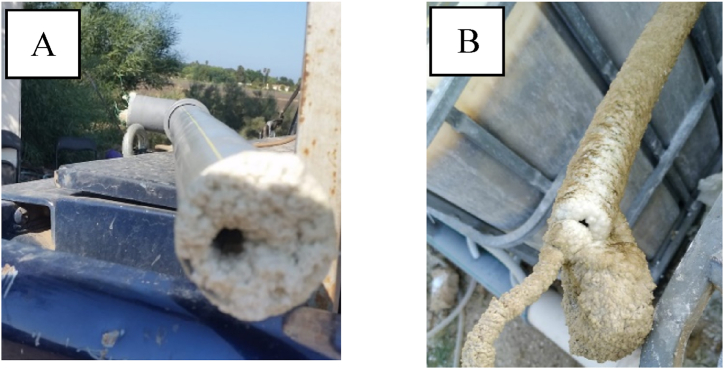
Mineral precipitation on (A) 2" outflow pipe and (B) 1.5 cm aeration hose, immersed in a fish tank receiving BWDC.

Visually, the control water was very different from the concentrate water, with the former being algae green, and the latter being tap-water clear. In the BWDC receiving tanks, significant mineral precipitation was observed on all submerged surfaces, including tank walls and pipes, which forced frequent scale removal. Fig. 2A and B depict a clogged 2" outflow pipe after less than 1 month of operation and a 1.5 cm clogged aeration hose after around 1 week of operation in a BWDC receiving tank, respectively. Such precipitation will likely pose a significant operational challenge under commercial application and was not observed at all in the SBWDC receiving and control tanks.

**Fig. 3:**
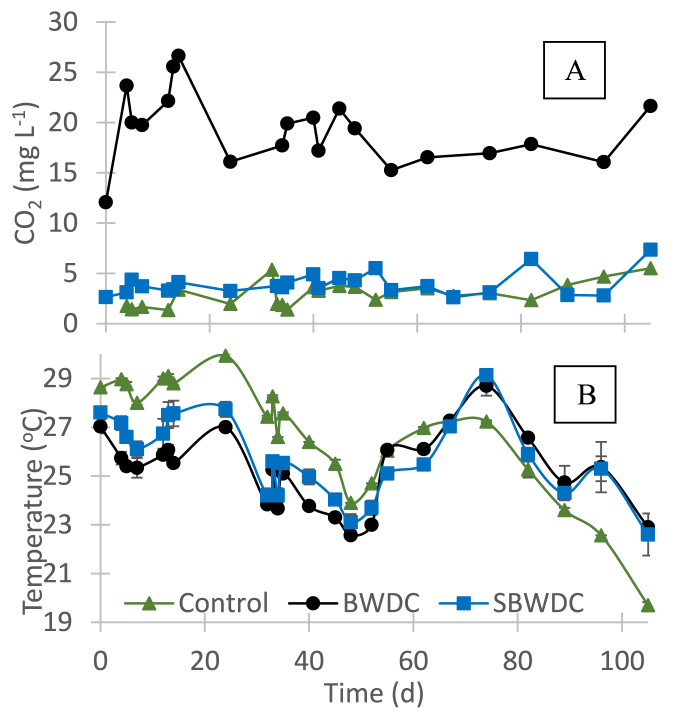
CO_2_ concentration (A) and water temperature (B) in the fish tanks.

Oxygen concentration in all fish tanks was above 5.7 mg L^−1^ throughout the experiment, well above the minimal recommended level of 5 mg L^−1^ [17]. The EC in both BWDC and SBWDC receiving tanks was virtually the same as in their feed water (Table 1), whereas the EC in the control tanks was 5.0 ± 0.2 dS m^−1^ throughout the experiment. The pH in all tanks varied between 7.7 and 8.0 throughout the experiment. These values were slightly higher than in the feed concentrate (Table 1), probably due to CO_2_ stripping upon fish tank aeration. Fig. 3 depicts the average CO_2_ concentration and water temperature in the fish tanks during the experiment.

**Fig. 4:**
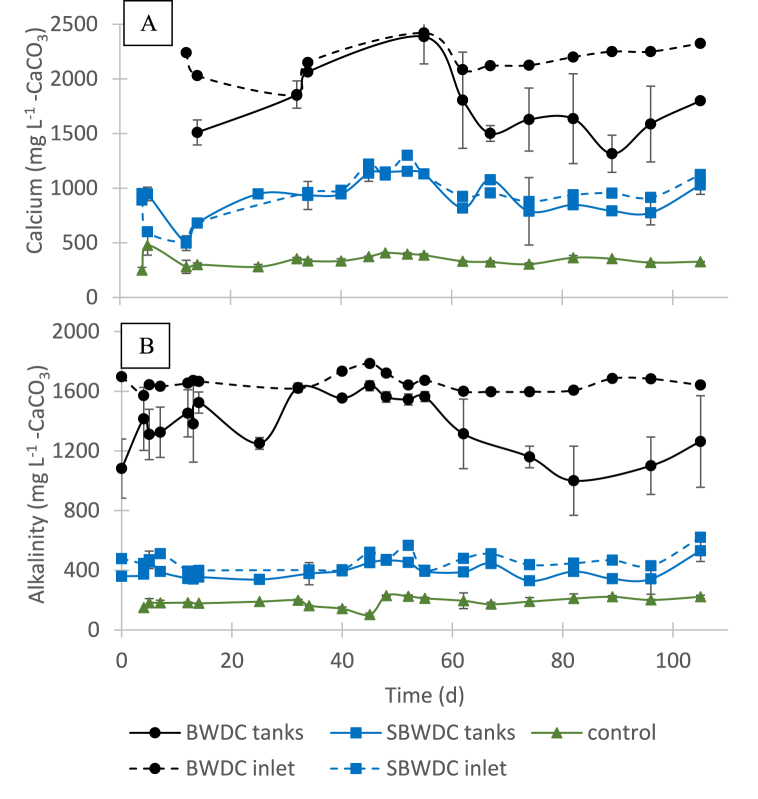
Average calcium (A) and alkalinity (B) concentrations in the fish tanks and in the inlets during the experiment.

Fig. 3A shows that CO_2_ concentration in the control fish tanks and in the SBWDC receiving fish tanks was similar and significantly lower than the threshold concentration range of 20–40 mg L^−1^ [14]. The CO_2_ concentration in the BWDC receiving fish tanks, however, was around 20 mg L^−1^. Since typical fish stocking density in commercial intensive systems may be two orders of magnitude higher than in the current study [18], CO_2_ concentration in intensive systems fed by BWDC may pose a serious issue. It is noted that CO_2_ concentration in the BWDC receiving tanks was lower than in their feed (Table 1) due to CO_2_ stripping in the fish tank. Fig. 3B shows that the temperature in the BWDC and in the SBWDC receiving tanks was nearly the same. Interestingly, the water temperature in the control tanks was higher by 2–4 °C than in the concentrate receiving tanks in the beginning of the experiment, but up to 3 °C lower by the end of the experiment. This was mainly attributed to the relatively constant temperature of the BWDC, and to the decrease in ambient temperatures during the experiment which started in midsummer and ended in mid-fall. Since fish growth rates are highly affected by temperature, these results reflect the benefit that can be attained when using BWDC in aquaculture, especially during winter. Fig. 4 depicts the average calcium and alkalinity concentration in the fish tanks and in their inlets during the experiment.

**Fig. 5:**
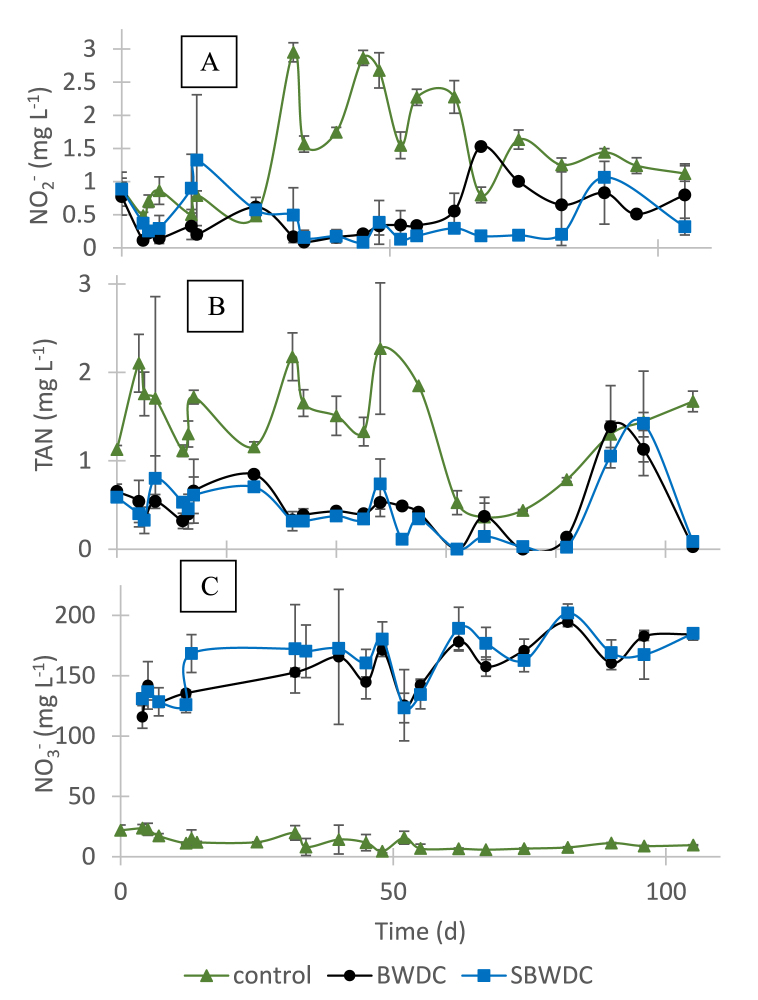
Average concentration of nitrite (A), TAN (B) and nitrate (C) in the fish tanks during the experiment.

Fig. 4 shows similar trends of calcium and alkalinity concentrations, suggesting that changes in calcium concentrations in the fish tanks, if any, followed Eq. (1). Fig. 4 also shows that calcium and alkalinity levels in the SBWDC receiving tanks and in their inlets were almost equal. However, calcium and alkalinity levels in the BWDC receiving tanks were up to 40% lower than in their inlets. This indicated that substantial CaCO_3_ precipitation occurred only in the BWDC receiving tanks, as anticipated and in agreement with the visual observations (Fig. 2). The precipitation of CaCO_3_ in these tanks varied with time, perhaps as result of aeration intensity change due to aeration hose clogging. This also explained the relatively high standard deviations observed in the corresponding tanks in Fig. 4A and B. Average concentrations of nitrogen species in the fish tanks are presented in Fig. 5.

Fig. 5A, B and 5C depict average concentration of nitrite, TAN and nitrate in the fish tanks, respectively. Fig. 5 shows that during most of the experiment, nitrite and TAN concentrations were relatively high in the control tanks and low in the concentrate receiving tanks, compared with recommended levels [15]. This was not surprising as the control water originated from an active separate fishpond, while the concentration of these species in the concentrate was very low (Section 3.1). On the other hand, nitrate concentration was relatively high in the concentrate receiving tanks, at typical values of 130–180 mg L^−1^ (30–41 mg N L^−1^), whereas nitrate concentration in the control tanks was 5–20 mg L^−1^ (1.0–4.5 mg N L^−1^) only. The relatively high nitrate level in the concentrate was associated with desalination of nitrate-rich groundwater at a relatively high recovery rate. Maximal acceptable nitrate levels for marine species are 20 mg N L^−1^ [19] and prolonged exposure to elevated nitrate concentrations were reported to decrease the immune response and increase mortality [20]. Nevertheless, nitrate levels in recirculating aquaculture systems (RAS) often exceed 90 mg N L^−1^ [21,22]. It was concluded that nitrate levels probably did not affect fish health in the concentrate receiving tanks but may pose a concern if RAS is used. In such cases, nitrate removal through processes such as algae cultivation or denitrification may be required.

### 3.3 Fish survival and growth

**Survival**. During acclimatization stage and in the first two weeks of the experiment no mortalities were recorded except for 1 fish in tank 5 (BWDC) and 1 fish in tank 8 (SBWDC). During days 15 and 16, massive fish mortality occurred in tanks 7 and 9 (SBWDC) only. The reason for this event was not clarified. However, during days 52–54, another similar mortality event occurred in tank 5 (BWDC) only, in which the dinoflagellate parasite *Oodinium* was visually detected only on the fish in that tank. It was presumed that this parasite was responsible for both events. Effective transmission of dinospores, such as of *Amyloodinium ocellatum*, are known to occur at water salinities above 10,000 mg L^−1^ [23]. The salinity of the concentrate (BWDC, SBWC) and control water was around 10,000 and 4,000 mg L^−1^, respectively, which may explain the disease outbreak in concentrate receiving tanks only. Approximately one week after the first mortality event, some fish from the control tanks (1–3) were transferred to tanks 7 and 9. The water in these tanks were mixed by control water for another week for acclimatization. Additionally, the bottom of all fish tanks was cleaned weekly thereafter, to prevent possible anaerobic bacterial activity. The experiment in tank 5 was discontinued after the second mortality event and survival and growth rates in all other fish tanks were considered only between days 34 and 105. The total number of fish in each water type at day 34 was 178, 153 and 153 in the control tanks, BWDC receiving tanks and SBWDC receiving tanks, respectively. Average respective survival rates between days 34 and 105 were 96%, 93.7% and 92.2%. These results indicated that European seabass could survive at high rates for at least 70 days in both BWDC and SBWDC operated at flow-through regime.

**Fig. 6:**
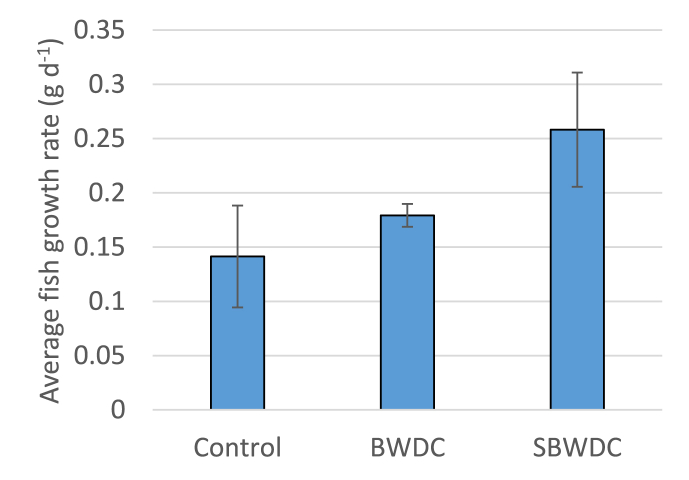
Average fish growth rates between days 48 and 108.

**Growth rate**. Fish growth rate was calculated in each tank between days 48 (1st fish weighing after the mortality event) and 105 (the end of the experiment). It is noted that on day 48, average fish weight (standard deviation) was similar in all tanks: 22.7 (2.6) g, 22.2 (1.6) g and 22.3 (2.1) g in the SBWDC, BWDC and control tanks, respectively. Average fish growth rate in each water type and corresponding standard deviation is depicted Fig. 6.

Fig. 6 shows that the highest average growth rate was in SBWDC and reached 0.26 g d^−1^. It was reported that growth rates of European seabass weighing 20 g and 40 g, in seawater at 26 ± 1 °C, were 0.25 g d^−1^ and 0.5 g d^−1^, respectively [24]. The somewhat lower growth rate in the SBWDC was attributed to its lower salinity, with respect to seawater. Although European seabass can adapt to very wide water salinities, its growth rate at 10 g L^−1^ water salinity (similar to BWDC) was reported to be 87% of the rate at 33 g L^−1^ (similar to seawater) [25]. As seen in Fig. 6, the average growth rate in SBWDC was 27% and 83% higher than in BWDC and in the control water, respectively. The lower growth rate in BWDC was associated with stress inflicted by the high calcium concentration, and perhaps also with CO_2_ supersaturation (Section 3.1). The lower growth rate in the control tanks was attributed to the lower salinity, as European seabass growth rate is known to increase with salinity [25]. It is also possible that fish cultivated in the control water experienced some stress associated with relatively high concentrations of TAN and nitrite (Fig. 5).

### 3.4 Pathological analysis

**Fig. 7:**
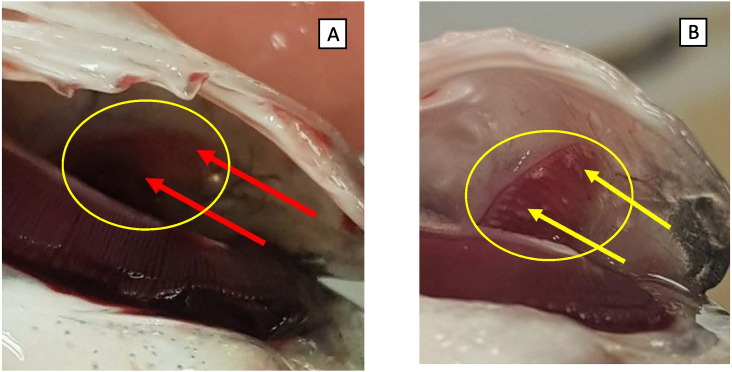
Pictures of gill cover's interior of fish from (A) SBWDC receiving tank (healthy) and (B) BWDC receiving tank (unhealthy).

No significant findings were observed in the fish that were examined at the beginning of the experiment. At the end of the experiment, all fish looked healthy and strong. In fish from all tanks, minor hyperplasia and very small presence of *Centrocestus formosanus* were observed in the gills as well as low presence of melanomacrophage centers in the spleen. These observations are not unusual in commercial aquaculture facilities and are of no concern. In the BWDC receiving tanks only, some injury appeared under the gill's cover, as shown in Fig. 7B, not observed in fish grown in SBDC, as shown in Fig. 7A. This injury was associated with calcium precipitation.

## 4 Implementation considerations

Kfar Masaryk fish farm was selected as a hypothetical model for large scale implementation assessment. The total fishpond water volume is roughly 2*10^6^ m^3^, while roughly 10^6^ m^3^ are left at the end of the summer due mainly to evaporation. The annual BWDC production of Kfar Masaryk desalination plant is also around 10^6^ m^3^. The fish farm is located between two brooks, the Hilazon and the Naaman, to which fishpond water can be discharged during winter as long as the monthly average discharge EC is under 5 dS m^−1^ and 10 dS m^−1^, respectively [8]. Since the EC of the existing fishpond water at the end of the summer is around 5 dS m^−1^, only the Naaman brook can be considered for disposal of the fishpond blended water, due to environmental regulations [8]. Four implementation options were assessed, taking into account water type and extensive (low fish density) or intensive (high fish density) cultivation method.

A. **Direct addition of BWDC to existing extensive fishponds.** The first option is to add raw BWDC directly into existing extensive fishpond. This option is the most straight forward but blending with existing fishpond water will result in relatively low water salinity (5–10 dS m^−1^) inadequate for cultivation of marine fish species such as gilthead seabream (*Sparus aurata*), assuming such species will behave like European seabass, and less favorable for cultivation of euryhaline species such as seabass (Section 3.3). In addition, the thermal energy of BWDC will be lost during winter due to the large fishpond surface and the risk of dinoflagellate parasites outbreak in the entire fish farm increases as salinity is elevated (Section 3.3).
B. **Extensive fish cultivation in fishponds fed by untreated BWDC only.** In this option, raw BWDC can be added to earthen fishpond, without blending with existing water, to grow fish extensively. Advantages include the ability of cultivating euryhaline and perhaps marine fish species, without BWDC pretreatment. Since aeration of such ponds is usually not needed, calcium precipitation is expected to be slow [13] and thus less affect fish growth. Approximately 500 tons of fish a year can be cultivated in this manner, assuming all the BWDC is utilized and fish density of 1 kg m^−3^. The main concern is that substantial amounts of CaCO_3_ will accumulate on the pond's floor. For example, at 50% of the potential 1.6 g L^−1^ calcium precipitation [13], around 800 tons of dry CaCO_3_ are expected to form annually. These precipitates, mixed with organics (fish excretion, dead protozoa, etc.), are expected to decrease pond's volume, and form large anaerobic zones at the datum. Removal and disposal of these precipitates may pose a serious operational and environmental issue. Stress associated with elevated CO_2_ concentration is also expected (Section 3.2).
C. **Intensive fish cultivation in fishponds fed by BWDC.** This option is similar to the previous one, only the fish are grown in intensive manner in raw BWDC. Advantages include no BWDC pretreatment (calcium removal) and the possibility of thermal energy utilization in small volume ponds, in which less heat is lost. However, substantial precipitation of CaCO_3_ in the system and subsequent maintenance issues may prohibit sustainable operation (Section 3.2). Fish injury (Section 3.4) and relatively slow growth rates due to the high CaCO_3_ and CO_2_ supersaturation (Section 3.3) are also plausible.
D. **Extensive and/or intensive fish cultivation in fishponds fed by SBWDC only.** In this option fish can be cultivated in treated BWDC in either extensive or intensive manner. Successful cultivation of euryhaline and perhaps of marine fish species, at adequate growth rates (Section 3.3) and without calcium precipitation issues (Sections 3.2–3.4), is anticipated. Preliminary BWDC softening in a designated facility can also reduce CO_2_ concentration (Sections 3.1 and 3.3) and allow CaCO_3_ recovery [13]. BWDC thermal energy can be utilized under intensive cultivation, where estimated annual production is around 500 ton, assuming characteristic fish density of 25 kg m^−3^ and 14% daily water exchange rate of aerated recirculating fishponds [26]. BWDC softening, however, requires certain capital and energy expenses. Interestingly, increasing fish stocking density and reducing water exchange rate are expected to reduce the extent of required softening. This is because a nitrification biofilter must be integrated in such systems to oxidize toxic ammonia. Nitrification adds acidity to the water thus reducing both pH and CaCO_3_ precipitation potential. Nitrification, however, also results in nitrate accumulation, which may become an issue since BWDC may already contain high nitrate concentration (Section 3.3). The effluents from intensive systems can be used for secondary fish cultivation in receiving extensive ponds, potentially doubling fish production.

## 5 Conclusions

European seabass was cultivated at over 92% survival rate for 70 days in both BWDC and SBWDC. Fish survival and growth rate in BWDC was acceptable but significant CaCO_3_ precipitation on submerged equipment and gill damage could prevent commercial application. Preliminary partial softening of BWDC increased fish growth rate, reduced risk of elevated CO_2_ concentrations, and eliminated intense mineral precipitation problems. The results indicated that preliminary partial softening of calcium rich BWDC can permit sustainable euryhaline fish cultivation. This approach may be implemented in locations where the disposal of growth water after harvest does not pose an environmental risk, e.g., directly into the sea or into floodwater stream during winter after blending with existing fishpond water. The risk of dinoflagellate parasite infection increases with salinity and should be considered. Combined fish cultivation in intensive and extensive systems fed by SBWDC is expected to result in maximal productivity and allow concentrate thermal energy utilization. Development of efficient softening system is crucial for the commercial implementation of the proposed approach.

## Author contribution statement

Sivan Klas: Conceived and designed the experiments; Performed the experiments; Analyzed and interpreted the data; Contributed reagents, materials, analysis tools or data; Wrote the paper.

Idan Rom: Performed the experiments.

Yakov Peretz: Conceived and designed the experiments; Performed the experiments.

## Data availability statement

Data included in article/supplementary material/referenced in article.

## Declaration of interest’s statement

The authors declare no conflict of interest.

## Declaration of competing interest

The authors declare that they have no known competing financial interests or personal relationships that could have appeared to influence the work reported in this paper.
